# Serological survey of *Toxoplasma gondii* infection in cats in Khon Kaen, Northeast Thailand

**DOI:** 10.14202/vetworld.2022.1779-1784

**Published:** 2022-07-25

**Authors:** Natthika Lakhamsen, Chalipa Chaisongkhram, Yanika Pattarasuplerk, Arayaporn Macotpet, Suvaluk Seesupa, Nitiwadee Lertitthikul, Pattaraanong Bupata, Panisara Kunkitti

**Affiliations:** 1 Faculty of Veterinary Medicine, Khon Kaen University, Khon Kaen 40002, Thailand; 2 Division of Medicine, Faculty of Veterinary Medicine, Khon Kaen University 40002, Thailand; 3 Division of Surgery, Faculty of Veterinary Medicine, Khon Kaen University 40002, Thailand; 4 Veterinary Teaching Hospital, Faculty of Veterinary Medicine, Khon Kaen University, Khon Kaen 40002, Thailand; 5 Division of Theriogenology, Faculty of Veterinary Medicine, Khon Kaen University 40002, Thailand

**Keywords:** adopted stray cat, feline, Khon Kaen, toxoplasmosis, zoonoses

## Abstract

**Background and Aim:**

Toxoplasmosis is a zoonosis caused by Toxoplasma gondii. Cats are known to be the definitive hosts that can excrete these environmentally resistant oocysts. Other mammals, avians, and even humans can serve as the intermediate host. T. gondii infection is often asymptomatic in healthy individuals; however, it could result in serious health problems in immunocompromised and pregnant individuals. This study investigated the occurrence of T. gondii infection in cats in Khao Suan Kwang and Mueang Khon Kaen.

**Materials and Methods:**

In total, 100 serum samples from cats, that is, 62 owned cats (31 males and 31 females) and 38 adopted stray cats (21 males and 17 females), were examined for antibodies against T. gondii through rapid immunochromatographic tests (ICT). Owners were asked to sign a consent form and answer the questionnaires before sample collection. Demographic information about the cats and their owners was also recorded.

**Results:**

The overall seroprevalence of cats positive for T. gondii antibodies was found to be 5%. Notably, the Toxoplasma antibody prevalence was significantly higher in the adopted stray cats (10.53% [4/38]) that roamed the zoo than in the owned cats (1.61% [1/62]) (p > 0.05). No significant difference was observed between male (8.33%) and female (1.92%) cats. The cat owners’ questionnaire revealed that more than half had never heard of toxoplasmosis before (67.7%), whereas 30.6% knew nothing about the disease transmission routes.

**Conclusion:**

This study presented a low seroprevalence of antibodies to T. gondii in owned cats from the Mueang Khon Kaen District, whereas high seroprevalence was detected in the adopted stray cats from Khao Suan Kwang. Adopted stray cats can have a higher potential for T. gondii infection; thus, they could be a source of toxoplasmosis transmission to humans. Therefore, it is essential to control the number of stray cats, and a screening test for antitoxoplasmosis could be recommended before adoption. Although the total seroprevalence was noted to be low, the zoonotic disease was present. Therefore, raising the community’s awareness and knowledge might reduce the disease transmission from animals to humans.

## Introduction

Toxoplasmosis is a zoonotic infection known to be caused by an obligate intracellular protozoan called *Toxoplasma gondii*. Domestic cats and members of the cat family (Felidae) are the definitive hosts, whereas mammals, avians, and even humans can serve as the intermediate hosts [1, 2]. *T. gondii* survives and sexually reproduces in the bodies of cats and parasitizes the bodies of other mammals through sexual and asexual reproduction. Notably, cats can be infected with *T. gondii* by eating contaminated food and water or from the environment. The infection will then replicate, survive in various tissues, and develop into oocysts [3]. Cats can shed oocysts in their feces 1–2 weeks after infection [4]. Most cats shed oocysts only after their first infection for a limited period; however, reinfection and shedding are possible [5, 6]. Most infected cats show no disease except when young or weak or have an immunodeficiency disorder. Humans can become infected through several routes, such as an infection from ingestion of cysts in uncooked meat, oocyst exposure by ingesting contaminated water and food [7], cleaning cat feces or litter box [8], and petting a cat that is already contaminated without washing hands before cooking or handling food. *T. gondii* can be transmitted across the placenta to the fetus in pregnant women and animals [9]. Its infection in a healthy person is asymptomatic; however, it can lead to severe symptoms and death in immunocompromised persons, such as those with HIV, patients with cancer receiving chemotherapy, people who have received an organ transplant, and those who are taking immunosuppressants [10]. Serological surveys are good indicators of *T. gondii* in cats and other species. However, several methods are available for detecting *T. gondii* antibodies. The methods include the Sabin–Feldman dye test, the enzyme-linked immunosorbent assay, and the latex agglutination test. Immunochromatographic tests (ICT) for detecting toxoplasmosis are of particular interest since they are of high sensitivity and specificity, simple, rapid, cost-effective, and easy to perform [11–14].

In Thailand, serological studies have shown widespread toxoplasmosis in many species. Several studies have previously reported seropositivity levels for *T. gondii*: 25% of pregnant women [15], 3.48%–18.7% of stray cats [16–19], 6.4% of pet cats, 18.84% of captive wild felids, 14.28% of free-range wild felids [18], 22% of dairy cattle [20], 25.6% of captive elephants [21], and 4.6% of rodents [16]. Various reports of *T. gondii* infection in humans also exist. Notably, seroprevalence can vary by population, region, and culture in Thailand [22]. The prevalence of *T. gondii* infection in healthy people ranging from 3.1% to 9.6% was reported in a Thai population [23, 24]. In 2013, the prevalence of *T. gondii* infection among women in Khon Kaen in Northeastern Thailand was only 2.6% [25]. However, a high prevalence was reported in the south of Thailand, showing 25% of pregnant women in Songkhla Province positive for anti-*Toxoplasma*.

Possible risk factors have been mentioned, such as exposure to unclean drinking water, consumption of undercooked meat, and a history of close contact with cats [15]. At present, cats are a popular choice for pets in Thailand because they are adorable and generally more affordable than dogs. The owners mostly obtained their pet cats from friends, cattery, or an adopted stray cat roaming near home. Interestingly, the number of stray cats in Thailand is astronomical and continues to increase. According to the Buddhist culture, people can benefit by feeding stray animals, whereas some people end up adopting them. Moreover, cat and theme cafés whose attraction is cats that can be watched and played with have become the new trend for people who love cats. Lack of awareness, poor sanitation, and personal hygiene might lead to infection if these cats have toxoplasmosis. Public places, such as cat cafés, could be disease reservoirs. However, its prevalence in cats in Khon Kaen province has not been studied, and the local people lack knowledge about this disease. Therefore, providing information and knowledge to the community might help people be aware of this disease to prevent disease transmission from cats to humans.

This study aimed to investigate the occurrence of *T. gondii infection* in cats in the Khon Kaen province.

## Materials and Methods

### Ethical approval

This study was approved by the Institutional Animal Care and Use Committee of Khon Kaen University, based on the Ethics of Animal Experimentation of the National Research Council of Thailand (Approval no. IACUC-KKU-25/64, Ref. no. 660201.2.11/110).

### Study period, location, and population

This study is a cross-sectional study from June 2019 to December 2020. Blood samples from 100 cats were collected from two different localities in Khon Kaen Provinces, Khao Suan Kwang district and Mueang district (for 38 and 62 samples, respectively). Cats older than 1 year of age were included in this study according to the previous study conducted by Inpankaew *et al*. [26] which found that cats aged 1–5 years had a higher prevalence of infection than cats aged <1 years. Before sample collection, the owner was asked to sign a consent form and answer the questionnaires.

###  Questionnaire

The questionnaire was answered by the owner, from which the following information was obtained: Owner’s level of education, gender and age of the cat, ownership and handling of cats, household water source, type of cat food, cat hygiene and personal hygiene habits, other pets, and toxoplasma knowledge.

### Sample collection

A sample of approximately 1 mL of blood was obtained from the cephalic, saphenous, or jugular vein with a 23G needle.

### Serological assay

In total, 10 μL of blood was drawn from the cephalic, saphenous, or jugular vein using a disposable plastic pipette. The whole sample volume (10 μL) and two drops of the buffer diluent (80–100 μL) were placed into sample window S of the test cassette. The test results were read 15 min after adding the buffer solution. Immunoglobulin G (IgG) against *T. gondii* was detected by rapid immunochromatographic technique using a commercial test kit (FASTest^®^ toxoplasma g, Megacore, United States) with a sensitivity and specificity of 98% and 97%, respectively. *T. gondii* antibodies in the sample migrated along the nitrocellulose membrane. They were bound to the fixed recombinant *T. gondii* antigens conjugated with gold particles forming a pink/purple-colored TEST line (T) according to the manufacturer’s instructions. The second pink/purple CONTROL line (C) indicates the correct test procedure.

### Statistical analysis

The infection rate of *T. gondii* infection was calculated from the proportion of positive serological tests from the total number of samples tested. The association between seropositivity and factors composed of gender (male or female), age (<5 or >5 years), and the category of cat (adopted stray cat or owned cat) was tested using the Fisher exact test, with p < 0.05 considered as statistically significant. The total number and percentage of cats and their owners are described in the demographic information.

## Results

### Serological survey of T. gondii infection in cats

*T. gondii* antibodies were detected in 5 (5%) of 100 cat blood samples. In total, 38 cats from Khao Suan Kwang district could be categorized as adopted stray cats since they were caught in the Khon Kaen Zoo area and raised by the zoo’s staff. In total, 4 (10.53%) of the 38 adopted stray cats were seropositive to antibodies of *T. gondii*. Meanwhile, of the 62 owned cats, only one cat (1.61%) was positive for *T. gondii* antibodies (Table-1).

**Table 1:** Detection of antibodies to *Toxoplasma gondii* in cats, in Khao Suan Kwang and Mueang Khon Kaen District, Thailand.

Categories	Number of positive/Number of sample	Percent positive
Adopted stray cats	4/38	10.53
Owned cats	1/62	1.61
Total	5/100	5.00

The proportion of male and female cats that tested positive for *T. gondii* was 8.3% (4/48) and 1.9% (1/52), respectively. The age of the cats was analyzed only for the owned cats (62), with no significant difference (p > 0.05) between the age groups (Table-2).

**Table 2:** Analysis of factors associated with Toxoplasma seropositive.

Factors	Number of positive/Number of sample (%)	p-value*
Gender		
Male	1/52 (1.9)	0.192
Female	4/48 (8.3)	
Age**		
<5 years	0/51 (0)	0.178
>5 years	1/11 (9.0)	
Categories		
Adopted stray cat	4/38 (10.5)	0.068
Own cat	1/62 (1.6)	

* Analysis by Fisher extract test.

** 38 adopted stray cats not included

Demographic information of cats and their owners showed that the typical seropositive cat is a male cat older than 5 years old that roamed inside and outside the house.

As per the findings of this study, it was found that more than half of the cat owners who participated in this study had never heard of toxoplasmosis (67.7%) before, and 30.6% had no knowledge about the disease’s transmission route. Moreover, 11% of the participants lived with pregnant women, 3.2% had kids, and 1.6% resided with immunocompromised persons (Table-3).

**Table 3:** Demographic information of cats and their owner retrieved from the questionnaire (owned cats, n = 62).

Characteristics	Number of infected cat per total cats (%)	No./total respondents (%)
Gender		
Male	1/31 (3.2)	
Female	0/31 (0)	
Age		
<5 years	0/51 (0)	
>5 years	1/11 (9.0)	
Cat’s living area		
Indoor	0/17 (0)	
Outdoor	0/26 (0)	
Both indoor and outdoor	1/19 (5.2)	
Cat’s defecation are		
Fixed location (have litter box)	1/53 (1.8)	
Anywhere	0/21 (0)	
Living with others animals		
Yes	1/41 (2.4)	
No	0/21 (0)	
Disease knowledge of the owner		
Know about toxoplasmosis before		
Yes		20/62 (32.2)
No		42/62 (67.7)
Know about the route of transmission		
Yes		19/62 (30.6)
No		43/62 (69.3)
Living with risk group		
Pregnant		7/62 (11.2)
Immunocompromised		1/62 (1.6)
Children		2/62 (3.2)

## Discussion

As per the findings of this study, it was determined that the seroprevalence of *T. gondii* infection among cats in Khon Kaen province was 5%. Our data correspond to the previous surveys of *T. gondii* infection in strays and owned cats in other regions in Thailand, previously reported to vary from 4.8% to 11%. Jittapalapong *et al*. [17] reported percentage seropositivity for *T. gondii* antibodies (4.8%) using the Sabin–Feldman dye test in stray cats in Bangkok and 6.4% in pet cats [27]. In addition, 3.48% (pet cats) and 11% (stray cats) were examined using latex agglutination technique [17]. The prevalence of *T. gondii* in cats was noted to vary depending on the type (stray or pet cat), age, environment, geography, and testing method. Our study also showed a low seroprevalence of antibodies to *T. gondii* (5%) in cats of the Khon Kaen Province. The main reason for the slow incidence of *T. gondii* in cats in the Khon Kaen Province in Thailand may be attributed to geography and climate, which is hot with intense sunlight. These conditions could affect the oocyst’s ability to sporulate. Oocysts lose their ability to sporulate when exposed to extreme conditions, freezing conditions (−21°C for a day or −6°C for 7 days) (50°C for 10 min, above 60°C for a few minutes), or extreme solar radiation [28, 29].

The study results highlight a significant difference in seroprevalence between the adopted stray cats (10.53%) and owned cats (1.61%) (p > 0.05). Most owned cats are either outdoor or semi-outdoor (kept indoors but allowed to roam freely outdoors). Cats can be infected by eating raw meat, eating or drinking contaminated food or water, or being exposed to a contaminated environment. Minimal exposure to these risk factors makes owned cats less likely to be infected than stray cats. Adopted stray cats in our study live in Khao Suan Kwang district next to Khon Kaen Zoo, which is a rural area far away from Mueang district, about 50 km. In the zoo, many animals can be intermediate hosts of *T. gondii*, such as sheep [30], goats [31], rusa deer [32], and other Felidae [33], among others. Furthermore, a high incidence of *T. gondii* infection in captive felids, that is, 15.4%–18.84%, was reported in Thailand [18, 34]. Stray cats can be infected through contaminated food or their environment. However, since these cats are predators, they can get infected by eating infected birds or rodents. In addition, stray cats roam while hunting, thus increasing the risk of potential exposure to *T. gondii*.

This study found that the seroprevalence of *T. gondii* infection in male cats was higher than in female cats at 8.33 (4/48) and 1.92 (1/52), respectively. Conversely, a study previously conducted in Bangkok discovered that the seroprevalence in female cats was higher than in male cats [16]. Notably, neither this study nor the previous study in Bangkok finds the gender of the cat to be significant. The seroprevalence of *T. gondii* infection in old cats (>5 years) was greater than that in young cats (<5 years), at 9.09 (1/11) and 0 (0/51), respectively, which is consistent with the findings of an earlier study conducted in Bangkok city [16]. Older cats have had more opportunities for exposure to the pathogen in their environment from going outside, hunting, and mating.

According to the authors, serological screening of toxoplasmosis in pet animals in Thailand has not been a practical routine until recently, especially in cats, most probably the definitive host of *T. gondii*. This finding might be due to the lack of concern about the disease, cost limitations, and limitations of laboratory facilities. Rapid ICTs are one of several serological methods that demonstrate the optimal analytical sensitivity and specificity for toxoplasma-IgG testing; ICT can detect *T. gondii* antibodies, particularly in chronic infections [11]. Compared to other tests, for example, the Sabin–Feldman dye test, which is expensive, requires a high level of laboratory biosecurity since it uses live tachyzoites as a source of antigen. ICT could be a good choice of serological method in screening for toxoplasmosis infection in cats in areas where toxoplasmosis is endemic. Our survey on toxoplasmosis knowledge of the cat owners showed that 48.53% (33/68) of our study participants had never heard of toxoplasmosis before. Alternatively, our online survey about toxoplasmosis knowledge of the cat owners showed that of 707 cat owners in Thailand, 57% (403/707) knew about toxoplasmosis, which suggests that around half of the cat owners know how to clean up cat feces and know about the prevention of the disease. Meanwhile, 43% (304/707) of cat owners (consisting of owners aged over 40 years, 32.89% (100/304) and 20.02% (161/304) of owners aged 35–40 years) have never heard of this disease, showing that the older people lack knowledge about toxoplasmosis more often than younger cat owners.

This study investigated the occurrence of *T. gondii* infection in cats for the first time in Mueang and Khao Suan Kwang districts, Khon Kaen Province in Thailand. The information from this study can reflect the situation of toxoplasmosis in the Khon Kaen region, create more awareness among people, and be used to study toxoplasmosis in the future. Additionally, establishing a restaurant or coffee shop where cats mingle with people could be another route of disease transmission. Therefore, our data from this study might help people understand toxoplasmosis and thus protect them from this zoonotic disease.

## Conclusion

Adopted stray cats have a higher potential to be infected by *T. gondii* than owned cats; thus, they could be a source of toxoplasmosis transmission to humans. Hence, it is essential to control the number of stray cats. Previously screening for toxoplasmosis before adopting stray cats might benefit the adopter. Although the seroprevalence of antibodies against *T. gondii* was low, this disease does exist. Therefore, as a preventive measure, people should be educated on how to protect themselves from infection. Remarkably, high-risk individuals such as pregnant women, immunodeficient persons, and cat owners can reduce their pets’ risk of exposure by keeping all cats indoors and avoiding feeding them raw meat.

The limitation of this study was the distribution of the samples. The samples in this study were collected from the Mueang and Khao Suan Kwang districts of Khon Kaen Province. Therefore, further studies with a larger sample size in additional sampling might help us see the overall epidemiological status of toxoplasmosis in the northeast region of Thailand.

## Authors’ Contributions

PK: Study design, methodology, writing, sample collection, and data collection. NL: Methodology, writing, and sample collection. CC: Methodology, writing, sample collection. YP: Methodology, writing, and sample collection. AM, NL, and KP: Sample collection. SS: Statistical analysis. PB: Laboratory work. All authors read and approved the final manuscript.
